# From Concept to Clinic: Pre‐Clinical Testing and Regulatory Considerations in Spine Implant Development

**DOI:** 10.1002/jsp2.70176

**Published:** 2026-04-14

**Authors:** Amit Benady, Jerrin Thadathil Varghese, Ryan David Quarrington, Morsi Khashan, B. Gangadhara Prusty, Ashish Diwan

**Affiliations:** ^1^ Department of Orthopedic Surgery Tel Aviv Medical Center Affiliated to Tel Aviv University Tel Aviv Israel; ^2^ Levin Center for Surgical Innovation Tel Aviv Medical Center Affiliated to Tel Aviv University Tel Aviv Israel; ^3^ Spine Labs, St George and Sutherland Clinical School University of New South Wales Sydney New South Wales Australia; ^4^ School of Mechanical and Manufacturing Engineering, UNSW Sydney New South Wales Australia; ^5^ ARC Centre for Automated Manufacture of Advanced Composites UNSW Sydney New South Wales Australia; ^6^ Center for Orthopedics and Trauma Research Adelaide Medical School, The University of Adelaide Adelaide Australia; ^7^ Department of Neurosurgery, Spine Unit Tel Aviv Medical Center Affiliated to Tel Aviv University Tel Aviv Israel; ^8^ Spine Service, Department of Orthopedic Surgery, St George and Sutherland Clinical School University of New South Wales Sydney New South Wales Australia; ^9^ Spinal Unit, Discipline of Orthopedic Surgery Royal Adelaide Hospital Adelaide South Australia Australia

**Keywords:** biomechanics, chemistry, manufacturing, and controls (CMC), Conformité Européenne (CE), design history file (DHF), European Union medical device regulation (EU MDR), finite element analysis (FEA), premarket approval, regulatory pathway, spine implant

## Abstract

**Background:**

Spinal implant development demands integration of biomechanical rigor with regulatory compliance. For surgeons, researchers, and biomedical engineers engaged in translational research, understanding the regulatory pathway, from the *Design History File* (DHF) through post‐market surveillance, is essential to smoothly transform an innovative idea into a safe and effective clinical product.

**Methods:**

This paper reviews the major regulatory frameworks governing spinal implants, including the *United States Food and Drug Administration* (FDA) pathways such as *Premarket Notification* [510(k)] and *Premarket Approval* (PMA), as well as the *European Union Medical Device Regulation* (EU MDR) leading to *Conformité Européenne* (CE) marking. International standards such as ISO 13485 for quality systems, ISO 14971 for risk management, ISO 10993 for biocompatibility, and ASTM mechanical testing standards are discussed. Particular attention is given to how biomechanics, including *Finite Element Analysis* (FEA), bench testing, and fatigue studies, are integrated into the pre‐market submission dossier.

**Discussion:**

Key elements of the regulatory process include design controls and documentation (DHF/technical file), *Chemistry, Manufacturing, and Controls* (CMC), preclinical validation through simulation, bench, cadaver, and animal testing, regulatory submissions across FDA and EU systems, and post‐market surveillance and lifecycle management. Common pitfalls involve overreliance on simulations without validation, inadequate risk management, and insufficient traceability. Emerging trends such as *in silico* trials, digital twins, and smart implants show promise, while global differences in classification, clinical requirements, and post‐market expectations highlight the ongoing challenge of regulatory divergence.

**Conclusion:**

Understanding the biomechanical foundations of the development process is crucial for the safe and successful translation of spine implants into clinical use. Surgeons and implant designers should engage early, understand the critical steps, and advocate for rigorous validation aligned with regulatory expectations to deliver safer and more effective implants to clinical practice.

## Graphical abstract

Spine implant development follows a regulated, iterative pathway from clinical need and design through material selection, prototyping, preclinical validation, and regulatory approval to post‐market surveillance. Biomechanical evaluation and risk‐based frameworks integrate across all stages, ensuring safe, effective translation from concept to clinical practice while enabling continuous improvement and next‐generation innovation.

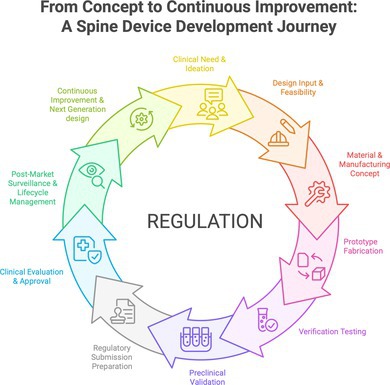

**Abbreviations**
510(k): Premarket Notification
ARTG: Australian Register of Therapeutic Goods
ASTM: American Society for Testing and Materials
CAPA: Corrective and Preventive Actions
CE: Conformité Européenne (CE marking)
CFR: Code of Federal Regulations
CMC: Chemistry, Manufacturing, and Controls (or Materials, Manufacturing, and Controls in device context)
CMMD: Custom‐Made Medical Device
CT: Computed Tomography
DHF: Design History File
EU MDR: European Union Medical Device Regulation
FDA: US Food and Drug Administration
FEA: Finite Element Analysis
HDE: Humanitarian Device Exemption
IDE: Investigational Device Exemption
IEC: International Electrotechnical Commission
IMDRF: International Medical Device Regulators Forum
IRB: Institutional Review Board (Also Ethics Committees or HREC—Human Research Ethics Committee)
ISO: International Organization for Standardization
MDD: Medical Device Directive (former EU directive, replaced by MDR)
MRI: Magnetic Resonance Imaging
MRL: Manufacturing Readiness Levels
OUS: Outside of United States
PEEK: Polyetheretherketone
PMA: Premarket Approval
PMDA: Pharmaceutical and Medical Device Agency
PMMD: Patient matched medical device
QMSR: Quality Management System Regulation
RCT: Randomized Controlled Trial
TGA: Therapeutic Goods Administration (Australia)

## 1 Introduction

Advances in materials, imaging, and biomechanics techniques have accelerated spinal implant innovation in recent decades [1, 2]. Yet regardless of novelty or promise, device translation into clinical practice depends on navigating a complex regulatory process. For surgeons and innovators, understanding this process is vital to ensuring patient safety and timely device availability. The development pathway for spinal implants lies at the intersection of biomechanical performance and regulatory requirements, where design innovation must align with compliance standards. Devices must not only withstand the mechanical demands of the spine, resisting fatigue, subsidence, and failure, which depend fundamentally on factors such as material selection, design, and geometry, but also demonstrate mechanical integrity and biological and imaging compatibility [3, 4]. Importantly, these devices must also satisfy strict regulatory requirements for safety and performance, risk management, and clinical evidence. These two dimensions are inseparable: biomechanical data underpin regulatory submissions, while regulatory frameworks define the standards against which that data is judged. Regulatory evaluation therefore begins with material justification grounded in biomechanical and biological criteria.

An example of this process is the introduction of polyetheretherketone (PEEK) cages [5]. Their radiolucency and Young's modulus, which is closer to that of bone than standard implants, made them attractive from a biomechanical perspective. However, the widespread adoption required regulatory approval based on fatigue testing, biocompatibility evaluation, and long‐term clinical follow‐up. Similarly, artificial discs (e.g., Charité Artificial Disc, Prestige, activeL or Mobi‐C) promised motion preservation, yet their path to approval demanded not only extensive bench testing but also large, prospective clinical trials under the Premarket Approval (PMA) pathway [6, 7]. These cases illustrate how even strong biomechanical concepts must be paired with robust safety and performance data (often comprising direct clinical evidence) before they can safely reach the operating room.

The regulatory landscape for orthopedic implants, particularly spine devices, involves complex pathways across multiple jurisdictions with varying evidence requirements and approval processes. One unique component of this landscape is the Custom‐Made Medical Device (CMMD) pathway, which provides a narrow exemption allowing manufacturers and surgeons to develop individualized, patient‐specific implants in rare clinical situations where no commercially available device can meet the patient's needs. Although these devices are exempt from formal premarket registration in many jurisdictions, they are not exempt from regulation; manufacturers and clinicians must still meet obligations relating to design justification, risk management, documentation, and post‐market vigilance [8]. This regulatory framework has evolved to balance innovation with patient safety while addressing the unique challenges posed by implantable medical devices.

**FIGURE 1:**
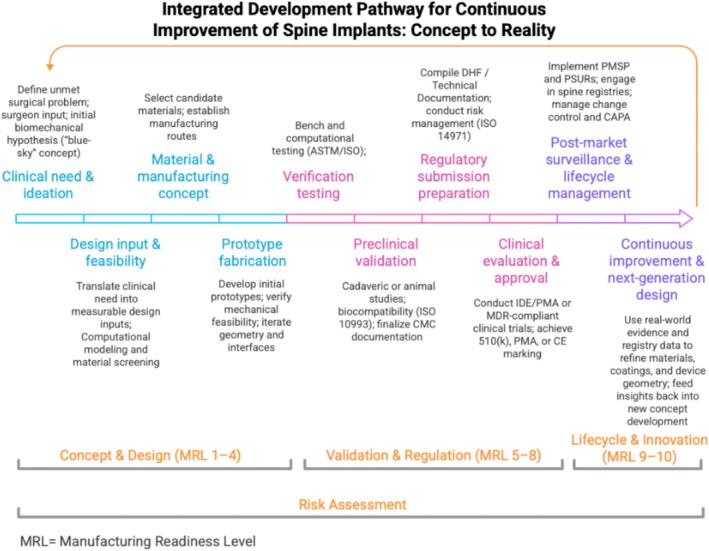
This figure illustrates the structured progression of spinal implant development from identifying a clinical need to continuous post‐market refinement. The pathway is divided into three main phases: Concept & Design (MRL 1–4), Validation & Regulation (MRL 5–8), and Lifecycle & Innovation (MRL 9–10). Each stage aligns with increasing Manufacturing Readiness Levels (MRL), encompassing concept generation, prototype fabrication, verification testing, regulatory preparation, clinical validation, and post‐market surveillance. The process emphasizes iterative feedback, risk management, and data‐driven improvement through registry evidence to inform implant design. Color information is best viewed in the online version.

The aim of this paper is to provide a streamlined, practice‐oriented roadmap for spinal implant development within the United States and European Union regulatory frameworks, which together encompass the largest global markets for orthopedic medical devices [9], highlighting the essential steps from concept through post‐market surveillance (Figure 1). By focusing on principles most relevant to orthopedic surgeons, researchers, and biomedical engineers, this paper emphasizes how pre‐clinical evaluation, clinical evidence, and regulatory compliance can work together to bring safer, more effective implants into clinical practice. The following section outlines how device risk classification determines the depth of evidence required, setting the stage for design controls and testing plans.

## 2 Regulatory and Standards Landscape

Medical devices are classified according to their level of risk, which determines the regulatory pathway and the amount of supporting evidence required (Table 1). Class I devices represent the lowest‐risk group, including simple tools such as bandages, stethoscopes, tongue depressors, and manual surgical instruments. In the *European Union under the Medical Device Regulation* (EU MDR 2017/745), Class I devices are considered low risk; however, sterile (Is), measuring (Im), and reusable surgical instruments (Ir) require limited conformity assessment by a Notified Body, which is an organization designated by the competent authority of an EU Member State to perform conformity assessment activities. Once designated, the Member State notifies the European Commission and other Member States of the Notified Body's authorization to evaluate device compliance, including verification of sterilization, measurement accuracy, and reprocessing procedures [10].

**TABLE 1:** Comparison of main regulatory pathways: FDA vs. EU MDR.

Aspect	FDA (United States)	EU MDR (Europe)
Device classification	Class I (low), II (moderate), III (high)	Class I, IIa, IIb, III
Primary pathways	510(k): substantial equivalence to predicate De Novo: novel low‐/moderate‐risk (Class I/II) PMA: Class III, clinical data required HDE: rare conditions	CE marking through conformity assessment by a notified body. Technical Documentation (Annex II/III) replaces Design Dossier Clinical Investigation per risk class
Evidence requirements	510(k): bench + biocompatibility	Class IIb and III: Require a clinical investigation and a Clinical Evaluation Report (CER).
	PMA: preclinical + clinical trials	
	De Novo: limited data depending on risk HDE: safety & probable benefit	
Post‐market requirements	Medical Device Reporting (MDR), recalls, Quality Management System Regulation (QMSR)	After CE marking, ongoing Post‐Market Clinical Follow‐up (PMCF) is mandatory to monitor long‐term safety and performance.
Outcome	FDA *clearance* (510(k)/De‐Novo) or *approval* (PMA/HDE)	CE marking for EU/EEA market access

Class II devices occupy the intermediate‐risk category and require more rigorous preclinical evidence to demonstrate safety and effectiveness. The US Food and Drug Administration (FDA) *Premarket Notification* (510(k)) pathway has dominated orthopedic device approvals, covering nearly 99% of submissions over the last 10 years, with 38% accounting for spine devices [11, 12]. For example, pedicle screw systems and interbody cages fall into Class II and are cleared through the FDA's 510(k) pathway by demonstrating substantial equivalence to a predicate device. Most 510(k) submissions emphasize mechanical testing rather than clinical trials, and the pathway generally requires limited clinical evidence compared to other approval routes [13, 14]. In the EU, these devices are often Class IIb, requiring a full *technical file* to be reviewed by a Notified Body.

Class III devices are considered the highest risk, as they are life‐supporting, life‐sustaining, or implanted long term, and their failure could result in serious harm. Spine implants that are novel in design, materials, or function, such as artificial discs or motion‐preserving dynamic stabilization systems, fall into this category. In the US, Class III devices must undergo *Premarket Approval* (PMA), which requires extensive bench and biocompatibility testing and carries a substantially greater evidence burden than 510(k) clearance [15]. In most cases, randomized controlled trials are necessary, and non‐inferiority study designs are also commonly employed [16, 17]. In Europe, Medical devices generally require establishing and maintaining comprehensive *technical documentation* (previously known as *design dossier*), and generally necessitate direct clinical evidence for spinal implants to demonstrate safety and performance before *Conformité Européenne* (CE) marking can be granted. For CE marking of spinal implants, a third‐party assessment is required by an independent notified body against EU Medical Device Regulations (Regulations EU 2017/745) [18]. Notified bodies are independent entities designated by EU member states and notified to the European Commission to carry out conformity assessment activities. In summary, Class II devices generally rely on extensive bench and biocompatibility data, while Class III devices require randomized clinical trials and closely monitored early clinical use (Table 2). These distinctions set the evidence expectations that guide subsequent design controls and testing strategies.

**TABLE 2:** Device classes and corresponding regulatory pathways.

Risk class	Typical device examples	FDA pathway	EU MDR pathway/oversight	Clinical evidence requirement
Class I (Low)	Surgical instruments, bandages	Exempt or 510(k) General Controls	Class I‐self‐certified; Notified body only for sterile (Is), measuring (Im), or reusable (Ir)	Not usually required
Class II (Moderate)	Pedicle screw systems, interbody cages	510(k): substantial equivalence; De Novo for novel designs	Class IIa or IIb‐conformity assessment via a notified body, Technical File review	Bench + limited clinical data
Class III (High)	Artificial discs, motion‐preserving implants	PMA: comprehensive preclinical and clinical trials	Class III‐Technical Documentation review; clinical investigation mandatory	Extensive clinical trials
Special/Rare	Humanitarian Use Devices (HUDs)	HDE: ≤ 8000 US patients/year	No direct MDR equivalent; handled as Class III	Safety & probable benefit

Across both the FDA and EU systems, evaluation is anchored by the International Organization for Standardization (ISO). For example, ISO 13485 defines requirements for a medical device‐specific quality management system [19], while ISO 14971 mandates a structured process for risk management throughout the product lifecycle [20]. The ISO 10993 family governs biological evaluation of medical devices and is essential for demonstrating that materials such as titanium, cobalt chrome, or PEEK are biocompatible and safe for implantation [21]. Both FDA and EU MDR require that manufacturers compile this information into structured documentation, known as the *Design History File* (DHF) in the US and the *technical file/documentation* in the EU. An early regulatory gap analysis helps identify potential deficiencies, such as missing risk analyses, insufficient fatigue testing, or lack of clinical evidence, before submission.

The FDA has also established additional pathways beyond the standard 510(k) and PMA processes, including the *De Novo* pathway for novel low‐ to moderate‐risk devices (Class I or II) and the *Humanitarian Device Exemption (HDE)* for rare conditions [22], (e.g., Ref [6]). The EU MDR emphasizes both premarket clinical investigation and post‐market follow‐up, while maintaining *Conformité Européenne* (CE) marking as the primary approval mechanism. Compared to the previous *Medical Device Directive* (MDD), the MDR introduced stricter requirements, with high‐risk and implantable devices now subject to particularly rigorous evaluation [23, 24].

Beyond the US and EU, Japan, Canada, Brazil, and Australia follow similar risk‐based frameworks aligned through the International Medical Device Regulators Forum (IMDRF) recommendations [25]. Australia's TGA [26], for example, accepts EU CE certificates for inclusion of a device in the Australian Register of Therapeutic Goods (ARTG), while TGA, Brazil, Japan's *Pharmaceutical and Medical Device Agency* (PMDA) [27] and Health Canada [28] accept *Medical Device Single Audit Program* [29] (MDSAP) audit reports as part of their regulatory framework for medical devices. These collaborations represent ongoing global harmonization toward consistent safety and performance expectation.

## 3 Pre‐Development and Design Controls

**FIGURE 2:**
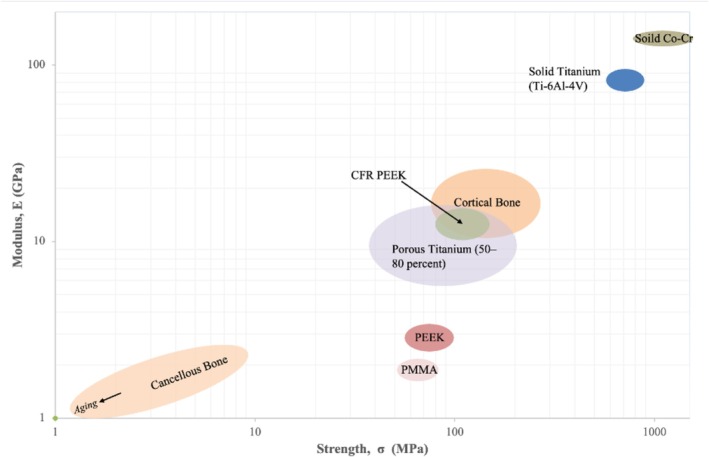
Ashby plot of strength versus modulus for common biomaterials used in orthopedic implants [30, 31, 32]. The diagram compares the elastic modulus (E) and strength (σ) of biological and engineering materials on logarithmic scales. Each colored oval represents the typical property range for a given material. Color information is best viewed in the online version.

The first step in developing a spinal implant is to translate the clinical problem and surgeon expectations into formal user requirements. These requirements are then expressed as design inputs, such as target stiffness, yield strength, and fatigue resistance. The resulting design outputs include detailed geometry, material choice (Figure 2), and interface features intended to meet those targets. At this stage, verification asks whether the device has been built correctly: finite element simulations, mechanical bench tests (axial compression, bending, torsion, fatigue), and dimensional checks all confirm that the design outputs match the design inputs. Validation, by contrast, asks whether the device itself is the right solution, demonstrated through cadaveric or animal testing and ultimately through clinical evaluation. Throughout development, every change and decision must be documented and traceable, with all supporting data compiled into a DHF under US FDA requirements or a technical documentation file under the EU MDR, linking simulations, experiments, and rationale across the project.

Evaluation of spinal and orthopedic implants often progresses through multiple phases, with the structure and depth of evidence adapted to device risk classification, intended use, and regulatory requirements (Table 3). Preclinical or Phase 0 work consists of extensive bench testing, cadaveric evaluations, and when relevant, animal studies. These experiments confirm mechanical integrity, surgical feasibility, and biological safety before any human implantation. Phase I trials then involve a small group of patients, focusing on short‐term safety, perioperative complications, and device performance in vivo. Phase II studies expand to a larger patient population, assessing preliminary efficacy endpoints such as fusion rates, implant stability, or motion preservation, while continuing to monitor safety. Phase III trials are large, multicenter, and often randomized, designed to compare the new implant against established devices or techniques, generating pivotal evidence for regulatory approval of high‐risk (Class III) implants. Finally, many devices undergo post‐market (Phase IV) surveillance, frequently through national or international registries, to capture long‐term durability, rare adverse events, and real‐world performance. Because verification and validation critically depend on the defined user requirements (and consequently the design solutions adopted, chosen materials and manufacturing routes), the next section specifies CMC decisions established before formal testing. Importantly, the requirements for clinical investigation are not uniformly structured according to the classical Phase I–IV terminology for medical devices in either the US or the EU. Rather, the extent of clinical evidence depends on factors such as device risk classification, design novelty, materials used, and the principal mode of action. For example, lumbar total disc replacement systems have required extensive prospective clinical investigations, including long‐term follow‐up data [7, 33, 34], prior to US market authorization, whereas less novel spinal implants may rely more heavily on predicate devices or existing clinical data [11].

**TABLE 3:** Phases of clinical evaluation for spinal and orthopedic implants.

Phase	Purpose	Typical study type	Key outcomes
Preclinical/“Phase 0”	Evaluate mechanical safety and feasibility before human use.	Bench testing (ASTM, ISO), cadaveric studies, animal models.	Fatigue strength, implant fixation, biocompatibility, cytotoxicity, sterilization, imaging safety and surgical feasibility.
Phase I: First‐in‐Human	Assess short‐term safety, procedural success, and device performance in a small patient group.	Early feasibility (typically ~10–20 participants).	Perioperative complications, early device function.
		Investigational Device Exemption (IDE) study will typically include this and Phase II/III below. Occasionally, OUS Phase I/II data may be used to file a full IDE with the FDA.	
Phase II: Pilot/Efficacy	Explore preliminary efficacy and refine indications while continuing to assess safety.	Prospective single‐arm or controlled study (typically ~50–100 participants).	Radiographic fusion rates, implant stability, motion preservation, adverse events.
Phase III: Pivotal Trial	Confirm safety and efficacy compared to standard of care; generate data for regulatory approval.	Multicenter randomized or controlled trial. Alternatively, a significant size cohort (hundreds to thousands of participants).	Clinical endpoints (pain, function), long‐term safety, non‐inferiority or superiority outcomes.
Phase IV: Post‐Market Surveillance	Evaluate real‐world performance, longevity, and rare complications after market entry.	Registries, observational studies, long‐term follow‐up. Reporting of AE's and SAE's form part of device surveillance.	Durability, adverse event trends, real‐world effectiveness.

## 4 Chemistry, Manufacturing, and Controls (CMC)

Building on the design inputs, CMC considerations are specified up front; selecting materials and defining manufacturing controls that set the mechanical, biological, and imaging performance to be confirmed in subsequent testing. Material choice is a decisive step in spinal implant development, as it governs biomechanical behavior, biological response, and imaging compatibility. Titanium alloys (Ti‐6Al‐4V) are widely used because of their excellent strength‐to‐weight ratio, corrosion resistance, and proven biocompatibility. Their modulus of elasticity, although still higher than bone, is closer to cancellous tissue compared to cobalt‐chromium alloys, reducing the risk of stress shielding [35]. Porous and surface‐treated titanium further enhance osseointegration, making it the workhorse of spinal fixation and fusion systems [36, 37]. Cobalt‐chromium alloys provide superior stiffness and fatigue strength, which is advantageous in demanding load‐bearing applications such as rods for deformity correction. However, their high modulus can concentrate forces at the bone‐implant interface, potentially increasing the risk of stress shielding and adjacent segment degeneration [38]. They also tend to produce more artifact on computed tomography (CT) and magnetic resonance imaging (MRI), which may limit postoperative evaluation [39, 40].

In recent years, PEEK has gained traction because of its radiolucency, allowing clear postoperative imaging and assessment of fusion. Its elastic modulus is closer to cortical bone, reducing subsidence and stress shielding. Carbon‐fiber–reinforced PEEK (CFR‐PEEK) modifies stiffness through fiber content and orientation, improving strength and reducing anisotropy, though higher carbon loading can introduce mild imaging artifacts on CT and MRI. However, PEEK is biologically inert, which can limit direct osseointegration [41]. To overcome this, modifications such as titanium coating, surface roughening, or composite formulations are increasingly employed. Eventually, hybrid constructs combine materials to leverage complementary strengths, for example, PEEK bodies with titanium endplates, or porous titanium inserts within polymer cages. These designs aim to balance radiolucency with bone integration and mechanical stability [5, 42, 43].

Beyond the base material, surface treatments, coatings, and porosity engineering play a pivotal role. Methods such as plasma spraying, electron beam melting, or grit blasting can improve bone on‐growth and mechanical interlock, while calcium phosphate or hydroxyapatite coatings further promote biological fixation [37, 44, 45]. While specific limits vary by implant type, reference standards include ISO 4287 for surface roughness, ASTM F2924 for additive manufacturing of titanium alloys [46], ASTM F2026 for the specification of PEEK polymers used in surgical implants [47] and FDA's guidance on additive manufacturing [48]. Regulatory authorities expect documented justification of these parameters within the DHF in the US or *technical file* in the EU. Process validation demonstrates repeatability and reliability, including sterilization and accelerated aging studies to confirm long‐term stability. Finally, process controls, including machining tolerances, additive manufacturing parameters, cleaning, and finishing, together with in‐process inspections and final release testing such as dimensional checks, mechanical sampling, and sterility verification, ensure that each device delivered to the surgeon is manufactured reproducibly and meets its intended specifications and safety profile.

## 5 Biomechanical, Biocompatibility, and Usability Assessment

Before a spinal implant can advance to clinical use, it must undergo rigorous preclinical validation to demonstrate mechanical integrity, biological safety, and reliability under physiologic conditions. Bench testing provides the cornerstone of evaluation, subjecting implants to static loading, cyclic fatigue, and combined load scenarios in line with international standards and regulatory guidance. The American Society for Testing and Materials (ASTM) developed widely recognized standards that specify mechanical testing methods for medical devices, including spine implants. Although these standards are not mandated in the EU, they are regarded as state‐of‐the‐art for testing spinal cages. Where available, ISO standards such as ISO 23089‐2:2021 for spinal interbody cages are used by regulatory authorities and manufacturers. There is a great deal of synergy between ISO and ASTM standards; for example, ISO 23089‐2:2021 cites a number of ASTM standards relevant to spinal implants. Mechanical testing represents a fundamental component of the approval process, with studies referencing specific standards for different device types. For example, ASTM F1717, F1798, and F543 apply to thoracolumbosacral pedicle screw systems [49], covering static and fatigue bending tests of spinal constructs, screw‐rod connection strength, and individual screw axial pullout performance, respectively. ISO 23089‐2:2021, ASTM F2077, and F2267 govern lumbar interbody fusion devices [50], defining static and dynamic compression, torsion, and shear testing as well as subsidence resistance using polyurethane foam blocks. ISO 12189, together with ASTM F2624, applies to dynamic spine implants [51] specifying flexion–extension fatigue and wear testing under simulated physiologic motion. ASTM F2346‐05, WK 4863, F1877‐98, F2423‐05, F2789‐10, and ISO/DIS 18192–1 address artificial disc replacement [7], focusing on range‐of‐motion durability, wear debris characterization, and long‐term cyclic endurance testing. Furthermore, Finite Element Analysis (FEA) plays an increasingly important role at this stage by predicting stress distribution, fatigue performance, implant subsidence, and bone‐implant interface behavior under simulated loading conditions [52]. However, FEA is typically useful for identification of the worst‐case design among different shapes and sizes of the implant. The required level of model accuracy depends on its context of use: when FEA is applied to screen design variants and identify a worst‐case configuration, the associated risk is relatively low and moderate accuracy may be acceptable; however, when it is used to define final device dimensions, the risk is substantially higher and a correspondingly higher level of model credibility is required. FEA results alone are unlikely to be sufficient to demonstrate safety and performance, especially in the context of dynamic loading. Hence, verification and validation should follow a tiered approach: model verification ensures numerical accuracy (mesh sensitivity, boundary‐condition stability), while validation compares simulation results with bench, animal, or cadaveric data. Quantitative agreement within defined tolerance bands is generally expected before simulation data can support regulatory evidence. Within this process, worst‐case scenario testing is essential, where boundary conditions, loading profiles, and device geometries most likely to induce failure are deliberately chosen.

By focusing on worst‐case scenarios, such as the smallest cage size or thinnest screw diameter, developers can ensure that safety margins are preserved even under the least favorable conditions, while sensitivity analyses probe how variations in material properties or implant positioning may further affect performance. Building on this, manufacturers must define safety factors and device margins that demonstrate the implant can withstand loads well beyond normal physiologic demands. This conservative approach provides confidence that the device will remain safe and functional throughout its intended lifespan, even in the face of patient variability and surgical technique differences.

Beyond mechanics, biocompatibility testing as outlined in the ISO 10993 series is required to confirm that implant materials are safe for long‐term human implantation. ISO 10993‐1 serves as the primary standard for biological evaluation [53], though additional cell and animal biocompatibility testing may be required for newer technologies, such as 3D‐printed PEEK implants [54]. Depending on the device and intended use, this testing may include cytotoxicity, sensitization, irritation, and long‐term implantation studies. For Class III devices requiring PMA, comprehensive biocompatibility data are mandatory.

In addition, human factors and usability engineering are now formal regulatory expectations for spinal implants. The FDA's *Applying Human Factors and Usability Engineering to Medical Devices* [55] guidance and the *standard International Electrotechnical Commission* (IEC) 62 366–1 both require manufacturers to implement a structured, risk‐based usability engineering process integrated with ISO 14971 risk management. This includes systematic identification of use‐related hazards, task and workflow analysis, evaluation of critical tasks, and iterative formative testing to refine instrument design, ergonomics, and labeling. Final validation must demonstrate that representative users can perform all critical steps safely and effectively under simulated clinical conditions, with residual use‐related risks reduced to acceptable levels. For spinal implants, where multistep instrumentation, constrained surgical fields, and high‐consequence manual tasks create substantial opportunity for use error, these frameworks ensure that human factors considerations are addressed with the same rigor as mechanical, biological, and preclinical testing.

## 6 Quality Systems and Manufacturing Compliance

Once materials and processes are defined, the reliability of production is ensured through a robust Quality Management System Regulation (QMSR). While the underlying principles of quality management, traceability, risk control, and continuous improvement are broadly harmonized internationally, regulatory authorities impose jurisdiction‐specific requirements. For example, a QMSR, typically aligned with ISO 13485, integrates directly with design controls, ensuring that every implant manufactured can be traced back to its design inputs and that deviations are captured systematically. This extends to facility and equipment validation, where manufacturing environments are monitored for contamination risks, and machinery is qualified to demonstrate consistent performance. Equally important is the control mechanisms in place for external partners: supplier qualification and outsourcing controls ensure that raw materials and subcontracted processes meet the quality specifications set by the legal manufacturer (for regulatory purposes) of the medical device. Finally, continuous improvement is maintained through change control procedures, Corrective and Preventive Actions (CAPA), and rigorous documentation practices. Although implementation details and inspection mechanisms differ between the FDA and EU notified bodies, these systems are functionally equivalent in intent. These mechanisms ensure that any identified nonconformity, whether during manufacturing, testing, or post‐market surveillance, triggers a structured response, minimizing risk to patients and ensuring regulatory compliance.

## 7 Regulatory Submission and Market Entry Strategy

The final stage of preclinical development is translating evidence into a regulatory submission that enables market access. In the United States, the choice of pathway depends on device risk and novelty: many spinal fixation systems and interbody cages qualify for the *Premarket Notification* [510(k)] process, where clearance is based on demonstrating substantial equivalence to a legally marketed predicate device. In this context, a predicate device is a previously FDA‐cleared spinal implant with the same intended use and similar technological characteristics, for example a PEEK lumbar interbody fusion cage already cleared for lumbar fusion procedures, against which the new device's design, materials, and mechanical performance are compared. The 510(k) route typically requires minimal clinical evidence, with substantial equivalence evidence serving as the primary requirement for clearance. Most submissions emphasize mechanical and biocompatibility testing rather than extensive clinical trials [11, 13, 14]. Novel technologies such as artificial discs or motion‐preserving implants often require PMA, which demands more extensive preclinical and clinical evidence [15]. PMA submissions generally mandate randomized controlled trials, sometimes designed as non‐inferiority or prospective studies for high‐risk devices [16, 17, 56]. For Class III spinal devices, and for certain grafts, rigorous clinical trial data are necessary, including Level I evidence [7, 57], with PMA submissions requiring prospective Investigational Device Exemptions (IDE)‐approved trials. Under EU MDR, clinical investigations must conform to ISO 14155:2020 (GCP for Medical Device Trials) and include a Clinical Evaluation Report (CER).

Briefly, when pursuing a 510(k), the ability to leverage predicate devices is central; establishing equivalence can significantly streamline the pathway by reducing the need for new clinical trials. For higher‐risk submissions, early interaction with regulatory bodies, through pre‐submission meetings or scientific advice workshops, can clarify requirements, reduce uncertainty, and align study designs with expectations. Finally, regulators place increasing emphasis on labeling, instructions for use, and human factors studies, recognizing that device safety depends not only on pre‐clinical studies but also on information provided with the implants and how they are used by surgeons.

In the European Union, manufacturers must compile a *technical documentation* under the EU MDR, reviewed by a notified body before CE marking can be granted. The technical documentation generally follows a structured format, encompassing mechanical test data, biocompatibility reports, risk management files, and clinical evaluation, along with labeling and usability evidence. MDR requirements emphasize both premarket clinical investigation and post‐market follow‐up, with registry data playing an increasingly important role in approvals [58, 59].

## 8 Post‐Market Surveillance, Change Control, and Lifecycle Management

Regulatory oversight does not end at market entry; implants must be monitored throughout their clinical lifespan to ensure ongoing safety and performance. Post‐market data collection occurs through national registries, surgeon‐reported outcomes, patient feedback/reporting, and mandatory adverse event reporting systems. This surveillance enables early detection of unexpected complications such as implant fracture, subsidence, or accelerated wear. When issues arise, manufacturers are obligated to initiate device vigilance measures, which can range from field safety notices and corrective actions to full product recalls, depending on the severity of risk. Equally important is change management. Even seemingly minor modifications, such as altering a coating process, changing suppliers, or adjusting geometry, can affect safety or performance. These changes must be formally assessed, documented, and, when necessary, submitted to regulators to demonstrate ongoing equivalence or to support a supplemental approval. Post‐market systems for spinal implants are governed by regional regulations, including 21 *Code of Federal Regulations* (CFR) Part 803 (Medical Device Reporting) in the United States and Articles 83–86 of the EU MDR. Manufacturers must maintain a Post‐Market Surveillance Plan (PMSP) and regularly issue Periodic Safety Update Reports (PSURs) addressing implant‐specific performance and adverse events. In the United States, national surveillance is increasingly supported by the American Spine Registry (ASR), a collaboration between the American Association of Neurological Surgeons (AANS) and the American Academy of Orthopedic Surgeons (AAOS). In both the United States and European Union, Unique Device Identification (UDI) systems further support post‐market surveillance, traceability, and change control by enabling device‐level tracking across manufacturing, clinical use, and adverse event reporting. Similar initiatives, such as the Australian Spine Registry and the British Spine Registry, collect longitudinal outcome data to support vigilance, trend analysis, and early signal detection. Together, these registries provide critical real‐world evidence that complements premarket testing and informs continuous regulatory evaluation.

## 9 Common Pitfalls, Practical Tips, and Recommendations

Despite advances in design and regulation, several recurring pitfalls continue to delay or derail spinal implant development. One of the most common is over‐reliance on finite element simulations without adequate validation, which may create unrealistic confidence in performance if not corroborated by bench or cadaveric testing [52, 60]. Another frequent weakness is poorly executed risk management and inadequate traceability, where design changes or hazard analyses are not fully documented, leaving gaps in the regulatory file. Finally, unanticipated design or manufacturing changes, such as changing materials, altering coatings, or updating suppliers, can trigger the need for new submissions if not anticipated and managed proactively.

To avoid these setbacks, implant developers/manufacturers should engage with regulators early and adopt modular documentation practices, ensuring that each stage of design and testing is clearly recorded and easily updated. A “design for regulatory” mindset from day one; treating verification, validation, and documentation as integral rather than peripheral tasks saves time and reduces the risk of rejection later. Above all, success depends on a structured test plan aligned with regulatory expectations, which defines the required simulations, bench tests, biocompatibility studies, and clinical evaluations in a logical sequence. This structured approach not only helps meet regulatory requirements but also gives surgeons confidence that the implant has been evaluated under realistic and demanding conditions.

## 10 Discussion

This review focuses on the challenge of translating biomechanical spinal implant concepts into clinical practice by integrating preclinical evaluation, clinical evidence, and regulatory requirements across major regulatory jurisdictions. Biomechanics is central to the safe and timely translation of spinal implants. FEA and advanced bench testing allow developers to predict failure modes and optimize designs before progressing to animal or clinical studies. When validated properly, these tools can shorten development timelines and reduce patient risk by ensuring only well‐characterized devices reach clinical trials. For surgeons, this means earlier access to implants that have already undergone rigorous mechanical scrutiny; for engineers, it highlights the need to link computational predictions with experimental and clinical outcomes.

Regulatory expectations differ across jurisdictions. In the United States, most spinal devices progress through the *Premarket Notification* [510(k)] pathway if a suitable predicate exists, whereas in the European Union under the MDR, equivalent devices still require substantial technical documentation and often clinical evidence. Despite harmonization efforts, regulatory divergence continues to create challenges for manufacturers. The US system's reliance on the 510(k) pathway contrasts with EU clinical investigation requirements. The MDR, which became fully applicable in 2021, has increased clinical evidence requirements compared to the previous MDD, and classification systems differ between jurisdiction [23]. Post‐market surveillance obligations also vary significantly, contributing to a complex compliance landscape for global market access [61].

Australia regulates medical devices through the *Therapeutic Goods Administration* (TGA), which uses a risk‐based classification system closely harmonized with the EU MDR. Many devices are included in the *Australian Register of Therapeutic Goods* (ARTG) on the basis of conformity assessment certificates issued by EU notified bodies. However, for high‐risk devices such as Class III implants, the TGA may require additional clinical evidence or conduct its own audits before ARTG inclusion [62]. This reliance on EU assessments streamlines access while maintaining oversight, but it also means that changes in EU regulations directly affect device approval pathways in Australia.

Looking forward, emerging trends are reshaping how evidence is generated and accepted. Regulatory agencies are beginning to consider in silico trials; computer‐based simulations of patient cohorts, as supplementary evidence, particularly when supported by validated FEA models. This approach could reduce the reliance on large animal studies or lengthy clinical trials for incremental innovations [63]. An even more transformative horizon is the integration of digital twins [64] and smart implants [65]. A digital twin is a computational replica of an individual patient's spine, continuously updated with clinical imaging and sensor data. By pairing implants with embedded sensors or adaptive materials, it becomes possible to monitor load, fusion progression, or implant micromotion in real time. Note that sensor‐integrated implants must comply with both device and electronic component regulations. Data acquisition and transmission are subject to International Electrotechnical Commission 60 601 (electrical safety) and ISO/IEC 27001 (cybersecurity) standards. Validation includes accuracy, drift, and biocompatibility of encapsulated sensors, ensuring that collected mechanical or physiologic data meet regulatory standards for clinical decision support. These smart implants could alert surgeons to complications earlier, guide rehabilitation, and generate personalized datasets that feed back into regulatory science. For biomedical engineers, the challenge will be creating validated models that regulators accept as reliable; for surgeons, the reward will be decision support systems that optimize outcomes for each patient.

Despite these advances, important challenges remain. Harmonization between jurisdictions is still incomplete, often forcing manufacturers to duplicate submissions across regulatory authorities. Global efforts to harmonize regulatory requirements are ongoing. ASTM International develops widely adopted mechanical and performance testing standards, while the ISO provides a global framework for device evaluation and quality management. The International Medical Device Regulators Forum (IMDRF) further promotes regulatory convergence across regions. Despite these initiatives, substantial differences remain between major markets, and innovation must consistently be balanced with patient safety. Greater harmonization has the potential to reduce barriers to global device development, while registry‐based post‐market surveillance is becoming an increasingly valuable tool for ensuring long‐term safety and effectiveness. Further, material innovations such as bioresorbable polymers, porous metals, and hybrid composites hold promise for improved osseointegration and load sharing, but they also raise new questions about long‐term performance and durability. Similarly, adaptive and sensor‐integrated implants may enhance monitoring and patient outcomes, yet they will require entirely new test standards and regulatory frameworks.

Finally, while regulatory approval determines whether a spinal implant is safe and performs as intended, it does not guarantee that the device will be reimbursed or widely adopted. Reimbursement decisions are made by government payers, insurers, and hospital procurement bodies, operate independently of regulatory pathways and focus on comparative clinical effectiveness, cost‐effectiveness, and budget impact. As a result, even devices that meet all regulatory requirements may face significant market‐access barriers if reimbursement codes are lacking, if payers deem the clinical benefits insufficient relative to cost, or if economic evidence has not been generated. These parallel processes mean that manufacturers must plan not only for regulatory compliance but also for health‐economic evaluation, coverage assessments, and coding strategies to ensure that an implant is both approvable and financially viable in clinical practice. Acknowledging these reimbursement challenges is therefore critical for understanding the full pathway from concept to widespread clinical adoption.

We acknowledge several limitations of this manuscript. This review provides a conceptual overview of how biomechanical evaluation and regulatory requirements intersect during spinal implant development. The discussion is focused on the United States and European Union frameworks and does not attempt to capture all jurisdiction‐specific variations or downstream considerations such as reimbursement or commercialization. Rather than cataloging every applicable standard or regulatory decision pathway, the manuscript highlights representative testing approaches and regulatory principles that commonly influence implant design and validation. Several simplifying assumptions are inherent to this approach. The regulatory pathways and testing strategies discussed are presented at a high level and may not fully reflect device‐specific nuances related to implant indication, anatomical region, patient population, or evolving regulatory guidance. In addition, the manuscript does not address detailed decision trees for regulatory resubmission, predicate selection, or clinical trial design, nor does it evaluate comparative effectiveness or long‐term clinical outcomes. These boundaries should be considered when interpreting the findings, which are intended to inform understanding of translational processes rather than to define device‐specific regulatory strategies.

## 11 Conclusions

The development of spinal implants requires an integrated pathway that unites biomechanical rigor with regulatory compliance. From material selection and design controls to preclinical verification and validation activities, regulatory submission, and post‐market surveillance, each step builds on disciplined documentation and transparent evaluation. For surgeon‐innovators, early engagement with both pre‐clinical testing and regulatory dimensions is essential: understanding how clinical needs translate into design inputs, how validation differs from verification, and how evidence is structured for approval accelerates efficient translation. With proactive planning, robust verification and validation, and continuous feedback from clinical practice, the journey from concept to clinic is not only feasible but can also lead to safer, more effective spine implants that address real surgical needs. Looking forward, increasing regulatory acceptance of validated *in silico* trials, registry‐based real‐world evidence, and emerging smart implants suggests a shift toward more data‐driven, adaptive regulatory frameworks. Ongoing global harmonization efforts through IMDRF and ISO are paving the way for integrated digital‐device ecosystems, where validated computational models, interoperable data standards, and real‐time post‐market analytics will increasingly bridge engineering innovation with regulatory science.

## Author Contributions

Conceptualization was performed by A.B., J.T.V., R.D.Q., M.K., B.G.P., and A.D. Methodology was developed by A.B., B.G.P., and A.D. Investigation and visualization were carried out by A.B., J.T.V., R.D.Q., M.K., B.G.P., and A.D. Supervision was provided by B.G.P. and A.D. A.B. drafted the original manuscript, and J.T.V., R.D.Q., M.K., B.G.P., and A.D. contributed to review and editing. All authors approved the final version of the manuscript.

## Conflicts of Interest

The authors declare no conflicts of interest.

## Data Availability

No new data were created or analyzed in this study. Data sharing is not applicable to this article.
